# Supplementary material for: Early Myocardial Strain Reduction and miR-122-5p Elevation Associated with Interstitial Fibrosis in Anthracycline-Induced Cardiotoxicity
Source: Biomedicines. 2024 Dec 27;13(1):45. doi: 10.3390/biomedicines13010045 (PMC11762338; doi:10.3390/biomedicines13010045)
Supplement: Supplementary file 1 [file biomedicines-13-00045-s001.zip › Supplemental Table 1.pdf]

Supplemental Table S1. In silico analysis of the target genes of hsa-miR-122-5p

ADAM10	*DR1*	*MASP1*	*RFXAP*
ADSS	*EPO*	*MGAT5*	*RIMKLA*
ALDOA	*ERBB4*	*MICU3*	*RIMS1*
ANKIB1	*FKBP5*	*MIPOL1*	*RPL17-C18orf32*
ANKRD13C	*FOXK2*	*MLLT1*	*SESN2*
ARFGEF1	*FOXO3*	*MMGT1*	*SLC12A6*
BACH2	*FOXP1*	*MOSPD1*	*SLC13A5*
BRPF1	*FOXP2*	*MYO9A*	*SLC25A34*
C18orf32	*FUNDC2*	*NDRG3*	*SLC41A1*
CANX	*G3BP2*	*NFAT5*	*SLC4A8*
CBL	*G6PC3*	*NICN1*	*SLC52A2*
CCAR1	*GALNT1*	*NPAS3*	*SLC7A1*
CCDC6	*GIT1*	*NPEPPS*	*SMARCD1*
CCDC97	*GNPDA1*	*NT5C3A*	*SMYD4*
CCNG1	*GPR107*	*OCLN*	*SOX6*
CCNYL1	*GRHL2*	*P4HA1*	*SP2*
CD320	*H1F0*	*PAK3*	*SPOCK2*
CDC42BPB	*HECTD3*	*PDE7A*	*SPRY2*
CLIC1	*HIF3A*	*PDK4*	*ST6GALNAC4*
CLIC4	*HNRNPU*	*PEG10*	*STX16*
CLIC5	*IGSF5*	*PIP4K2A*	*SUCLA2*
CPEB1	*IHH*	*PKM*	*TBC1D10B*
CS	*IL1RN*	*PLAG1*	*TBC1D22B*
CTDNEP1	*KCNJ13*	*PLEKHB2*	*TBR1*
DDR2	*KDELC2*	*PRKRA*	*TFDP2*
DEDD	*KIF5B*	*PTPRB*	*USP53*
DICER1	*LAMC1*	*PXMP4*	*VAMP3*
DLAT	*LRP10*	*RBM47*	*ZBTB41*
DLG2	*MAF1*	*RBP5*	*ZNF827*

In silico analysis of the predicted target genes in miRDB and Target Scan databases. *ADAM10*: ADAM metallopeptidase domain 10; *ADSS*: adenylosuccinate synthase; *ALDOA*: aldolase fructose-bisphosphate A; *ANKIB1*: ankyrin repeat and IBR domain containing 1; *ANKRD13C*:

ankyrin repeat domain 13C; *ARFGEF1*: ADP ribosylation factor guanine nucleotide exchange factor 1; *BACH2*: BTB domain and CNC homolog 2; *BRPF1*: bromodomain and PHD finger containing 1; *C18orf32*: chromosome 18 open reading frame 32; *CANX*: calnexin; *CBL*: Cbl proto-oncogene; *CCAR1*: cell division cycle and apoptosis regulator 1; CCDC6: coiled-coil domain containing 6; *CCDC97*: coiled-coil domain containing 97; *CCNG1*: cyclin G1; *CCNYL1*: cyclin Y like 1; *CD320*: CD320 molecule; *CDC42BPB*: CDC42 binding protein kinase beta; *CLIC1*: chloride intracellular channel 1; *CLIC*: chloride intracellular channel 4; *CLIC5*: chloride intracellular channel 5; *CPEB1*: cytoplasmic polyadenylation element binding protein 1; *CS*: citrate synthase; *CTDNEP1*: CTD nuclear envelope phosphatase 1; *DDR2*: discoidin domain receptor tyrosine kinase 2; *DEDD*: death effector domain containing; *DICER1*: dicer 1, ribonuclease III; *DLAT*: dihydrolipoamide S-acetyltransferase; *DLG2*: discs large MAGUK scaffold protein 2; *DR1*: down-regulator of transcription 1; *EPO*: erythropoietin; *ERBB4*: erb-b2 receptor tyrosine kinase 4; *FKBP5*: FKBP prolyl isomerase 5; *FOXK2*: forkhead box K2; *FOXO3*: forkhead box O3; *FOXP1*: forkhead box P1; *FUNDC2*: FUN14 domain containing 2; *G3BP2*: G3BP stress granule assembly factor 2; *GALNT1*: polypeptide N-acetylgalactosaminyltransferase 1; *GIT1*: GIT ArfGAP 1; *GLCE*: glucuronic acid epimerase; GLUL glutamate-ammonia ligase; *GNG13*: G protein subunit gamma 13; *GNPDA1*: glucosamine-6-phosphate deaminase 1; *GPR107*: G protein-coupled receptor 107; *GRHL2*: grainyhead like transcription factor 2; *H1F0*: H1 histone family member 0; *HECTD3*: HECT domain E3 ubiquitin protein ligase 3; *HIF3A*: hypoxia inducible factor 3 subunit alpha; *IGSF5*: immunoglobulin superfamily member 5; *IHH*: Indian hedgehog signaling molecule; *IL1RN*: interleukin 1 receptor antagonist; *KCNJ13*: potassium voltage-gated channel subfamily J member 13; *KDELC2*: KDEL motif containing 2; *KIF5B*: kinesin family member 5B; *LAMC1*: laminin subunit gamma 1; *LRP10*: LDL receptor related protein 10; *MAF1*: MAF1 homolog, negative regulator of RNA polymerase III; *MASP1*: mannan binding lectin serine peptidase 1; *MGAT5*: alpha-1,6-mannosylglycoprotein 6-beta-N-acetylglucosaminyltransferase; *MICU3*: mitochondrial calcium uptake family member 3; *MIPOL1*: mirror-image polydactyly 1; *MLLT1*: MLLT1, super elongation complex subunit; *MMGT1*: membrane magnesium transporter 1; MOSPD1: motile sperm domain containing 1; *MYO9A*: myosin IXA; *MYOCD*: myocardin; NDRG3: *NDRG* family member 3; *NFAT5*: nuclear factor of activated T cells 5; *NICN1*: nicolin 1; *NPAS3*: neuronal PAS domain protein 3; *NPEPPS*: aminopeptidase puromycin sensitive; *NT5C3A*: 5'-nucleotidase, cytosolic IIIA; *OCLN*: occludin; *P4HA1*: prolyl 4-hydroxylase subunit alpha 1; *PAK3*: p21 (RAC1) activated kinase 3; *PDE7A*: phosphodiesterase 7A; *PDK4*: pyruvate dehydrogenase kinase 4; PEG10: paternally expressed 10; *PIP4K2A*: phosphatidylinositol-5-phosphate 4-kinase type 2 alpha; *PKM* pyruvate kinase M1/2; *PLAG1*: PLAG1 zinc finger; *PLEKHB2*: pleckstrin homology domain containing B2; *PRKRA*: protein activator of interferon induced protein kinase EIF2AK2; *PTPRB*: protein tyrosine phosphatase, receptor type B; *PXMP4*: peroxisomal membrane protein 4; *RBM47*: RNA binding motif protein 47; *RBP5*: retinol binding protein 5; *RFXAP*: regulatory factor X associated protein; *RIMKLA*: ribosomal modification protein rimK like family member A; *RIMS1*: regulating synaptic membrane exocytosis 1; *RPL17-C18orf32*: RPL17-C18orf32 readthrough; *SESN2*: sestrin 2; *SLC12A6*: solute carrier family 12 member 6; *SLC13A5*: solute carrier family 13 member 5; *SLC25A34*: solute carrier family 25 member 34; *SLC41A1*: solute carrier family 41 member 1; *SLC4A8*: solute carrier family 4 member 8; *SLC52A2*: solute carrier family 52 member 2; *SLC7A1*: solute carrier family 7 member 1; *SMARCD1*: SWI/SNF related, matrix associated, actin dependent regulator of chromatin, subfamily d, member 1; *SMYD4*: SET and MYND domain containing 4; *SOX6*: SRY-box 6; *SP2*: Sp2 transcription factor; *SPOCK2*: SPARC (osteonectin), cwcv and kazal like domains proteoglycan 2; *SPRY2*: sprouty RTK signaling antagonist 2; *ST6GALNAC4*: ST6 N-acetylgalactosaminide alpha-2,6-sialyltransferase 4; *STX16*: syntaxin 16; *SUCLA2*: succinate-CoA ligase ADP-forming beta subunit; *TBC1D10B*: TBC1 domain family member 10B; *TBC1D22B*: TBC1 domain family member 22B; *TBR1*: T-box, brain 1; *TFDP2*: transcription factor Dp-2; *USP53*: ubiquitin specific peptidase 53; *VAMP3*: vesicle

associated membrane protein 3; *ZBTB41*: zinc finger and BTB domain containing 41; *ZNF827*: zinc finger protein 827
